# Age-related changes in time perception: The impact of naturalistic environments and retrospective judgements on timing performance

**DOI:** 10.1177/17470218211023362

**Published:** 2021-06-05

**Authors:** Martin Riemer, Thomas Wolbers, Hedderik van Rijn

**Affiliations:** 1 Department of Experimental Psychology, University of Groningen, Groningen, The Netherlands; 2 Aging & Cognition Research Group, German Center for Neurodegenerative Diseases (DZNE), Magdeburg, Germany; 3 Center for Behavioral Brain Sciences (CBBS), Magdeburg, Germany

**Keywords:** Time perception, retrospective, prospective, aging, ecological validity

## Abstract

Reduced timing abilities have been reported in older adults and are associated with pathological cognitive decline. However, time perception experiments often lack ecological validity. Especially the reduced complexity of experimental stimuli and the participants’ awareness of the time-related nature of the task can influence lab-assessed timing performance and thereby conceal age-related differences. An approximation of more naturalistic paradigms can provide important information about age-related changes in timing abilities. To determine the impact of higher ecological validity on timing experiments, we implemented a paradigm that allowed us to test (1) the effect of embedding the to-be-timed stimuli within a naturalistic visual scene and (2) the effect of retrospective time judgements, which are more common in real life than prospective judgements. The results show that compared with out-of-context stimuli, younger adults benefit from a naturalistic embedding of stimuli (reflected in higher precision and less errors), whereas the performance of older adults is reduced when confronted with naturalistic stimuli. Differences between retrospective and prospective time judgements were not modulated by age. We conclude that, potentially driven by difficulties in suppressing temporally irrelevant environmental information, the contextual embedding of naturalistic stimuli can affect the degree to which age influences the performance in time perception tasks.

## Introduction

Time perception is a fundamental aspect of everyday life and undergoes various changes across the lifespan (Löckenhoff & Rutt, 2015). Several studies reported reduced timing abilities in older adults (Block et al., 1998; Mioni et al., 2020; Turgeon et al., 2016), and age-related changes in the perception of time are currently discussed as a potential cognitive marker for Alzheimer’s disease and other forms of dementia (El Haj & Kapogiannis, 2016; Maaß et al., 2019; Rueda & Schmitter-Edgecombe, 2009). However, most timing studies implement highly artificial stimuli, and it is unknown to which degree the measured changes in timing performance are specific to the abstract nature of typical lab-based experiments, questioning the external validity of interval timing tasks (Boltz, 2005; Matthews & Meck, 2014; van Rijn, 2018). Specifically, in lab experiments, participants are often asked to judge the duration of stimuli presented outside of any context (e.g., Riemer et al., 2019), whereas timed events in real life are always occurring within a specific context. The well-known red phase of a traffic light—often referred to as exemplifying the ubiquity of timing behaviour in real life—is always experienced within a rich visual scenery, and it is unclear how this potentially distracting information affects the timing process. The impact of irrelevant contextual information is especially important with respect to age-related changes in time perception because older adults often exhibit difficulties to suppress irrelevant information (Gazzaley et al., 2005; Lustig et al., 2007).

Every experience involves a temporal aspect (i.e., a duration) and our ability to perceive, recognize, and discriminate between different durations developed through our experience within complex and rich environments. For example, during a walk in the neighbourhood, we might observe other people engaged in various activities, cars halting for a moment before driving on, we might hear the sound of a church bell, and so on. In lab experiments, however, such complex, situated events are often replaced by meaningless, abstract stimuli that are presented outside of a common context. Although this approach has clear advantages with respect to the controllability of stimuli, it often lacks ecological validity, and the results might not be generalisable to real-world timing (Boltz, 2005; Matthews & Meck, 2014; van Rijn, 2018). The most minimal way to test for a potential effect of irrelevant contextual information consists in the implementation of a naturalistic scene in which the stimuli are embedded. The use of more realistic stimuli has been proposed earlier (Riemer et al., 2018; Roseboom et al., 2019; Schlichting et al., 2018; van Rijn, 2014), and a recent study suggested that the temporal context effect—an effect that is associated with memory impairments (Maaß et al., 2019)—is more pronounced when probed with naturalistic compared with abstract stimuli (Maaß et al., 2021).

Another difference between real-world timing and lab experiments consists in the prevalence of prospective timing studies, that is, participants are aware of the time-related nature of the experiment and are explicitly instructed to attend to the duration of stimuli (Block & Zakay, 1997). This is a substantial deviation from natural timing behaviour, as we hardly ever deliberately focus our attention to the passage of time. Subjectively reported difficulties in time perception almost always refer to retrospective timing, that is, when someone estimates the duration of past events or periods without specifically having paid attention to its temporal aspects (e.g., Coelho et al., 2016). For example, we do not explicitly monitor elapsed time when waiting for a webpage to load, we just eventually feel that it takes too long. Thus, retrospective time judgements depend more on implicit, automatic attention, whereas prospective time judgements rely more on deliberately guided attention. Some studies suggest that the ability to deliberately orient attention to specific aspects of the task is preserved in older adults (Chauvin et al., 2016; Heideman et al., 2018), but when such an attentional focus is not specifically instructed, as in retrospective tasks, age-related changes in timing behaviour might become apparent. We therefore assume an advantage for retrospective tasks to detect age-related changes in timing behaviour. In a similar way, the requirements for memory processes also differ between retrospective and prospective judgements. Only in prospective tasks, explicit memorisation is possible (e.g., by verbal iterations), whereas retrospective tasks rely on automatic consolidation processes alone.

Nevertheless, studies implementing retrospective time judgements are scarce, most likely due to a substantial constraint they are confronted with: Studies on retrospective timing are usually limited in the number of trials per participant because the time-related nature of the experiment is revealed after the first trial, and participants will inevitably change their attentional focus to temporal aspects in the following trials (Matthews & Meck, 2014).

The objective of this study is twofold. First, we investigated the degree to which age-related differences in time perception are affected by the stimulus material (i.e., whether the to-be-estimated durations are demarcated by events occurring in a naturalistic scene or by a sequence of object images outside of any context). Second, as changes in timing behaviour in older adults might be influenced by memory deficits (Maaß et al., 2019) and attention processes (Lustig, 2003), we examined whether age-related differences in time perception are more pronounced for retrospective compared with prospective judgements.

In this study, we repeatedly presented our participants with a visual naturalistic scene, in which several events occurred (e.g., street light illuminates, headlights of a car turns on). Each event had a specific duration. We informed the participants to just pay attention to the identity of the presented events, withholding the specific information that duration was the critical feature they would have to judge later during the study. General attention towards the stimuli was ensured by intermediate recognition tasks, in which target and lure events were presented (e.g., “Did you see this event happening?”). After the events had been learned, the time-related nature of the experiment was revealed and participants were asked to retrospectively judge the durations of the events.

**Figure 1.**
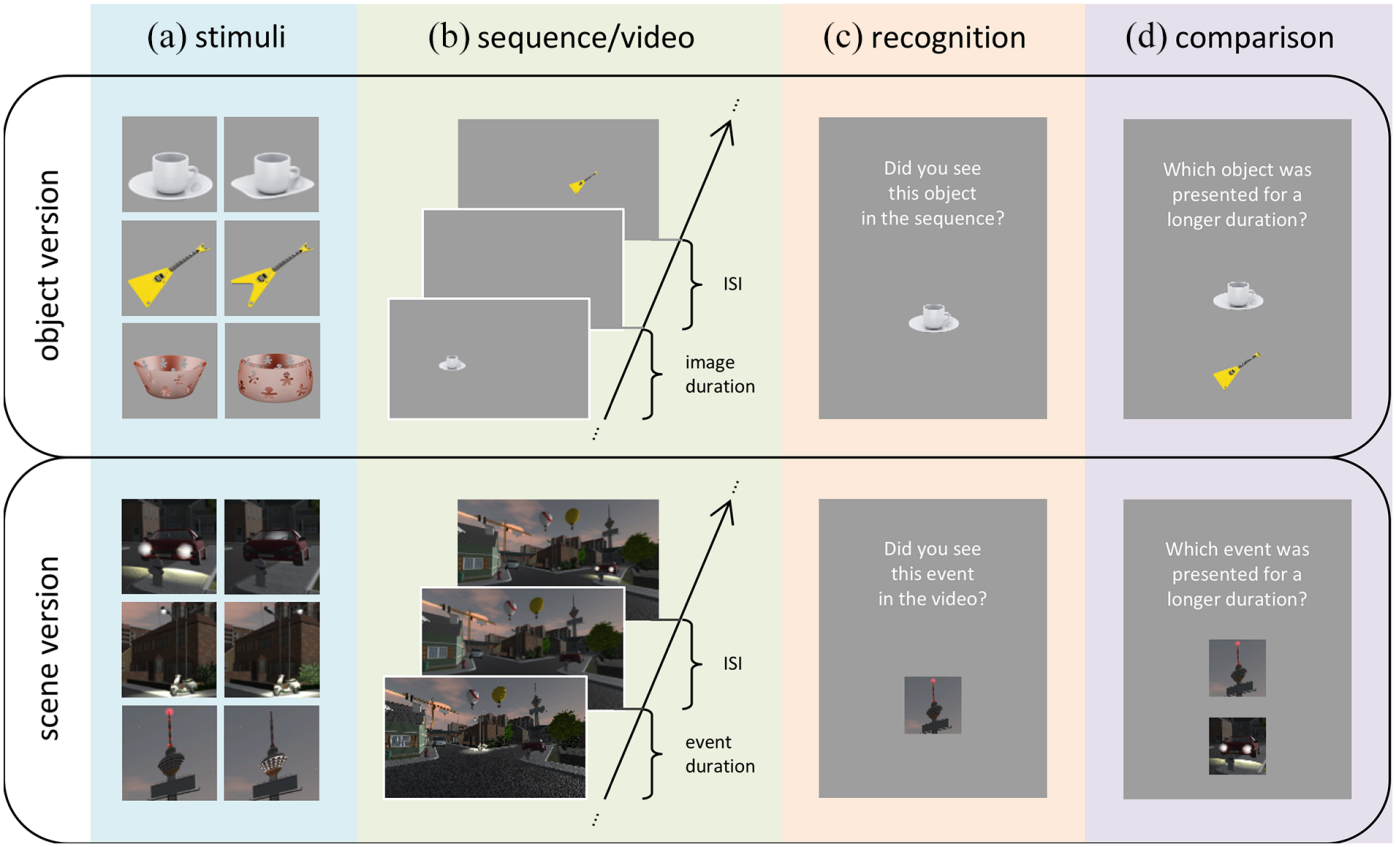
Pairs of target and lure stimuli (a) and experimental blocks (b to d) for the object version, implementing a sequence of object images (top), and the scene version, using a video of a naturalistic urban scene (bottom). During the encoding phase, (b) 10 object images (or events within a visual scene) were presented for different durations and at different locations, followed by (c) a recognition task, in which the participants judged whether an object/event appeared in the sequence/video. (d) For retrospective and prospective time judgements, participants judged which of two objects/events was presented for a longer duration.

To interpret timing performance, three contrasts were implemented. First, to relate the performance to a more abstract version of the same paradigm, we tested a parallel version in which object images (cf. Figure 1a) outside of a common scenic context were presented as duration stimuli. Second, to relate retrospective judgements to prospective ones, participants performed the judgements a second time (i.e., after they were presented again with the stimuli). Third, to relate the incremental benefit of prospective time judgements (relative to retrospective judgements) to that of another domain, we also implemented retrospective and prospective judgements about the spatial location of the events/stimuli.

Based on the notion that timing behaviour in older adults might be influenced by difficulties to suppress irrelevant information (Gazzaley et al., 2005; Lustig et al., 2007) and, especially when the immediate relevance of timing is unknown, by affected attention and memory processes (Lustig, 2003; Maaß et al., 2019), we hypothesised that age-related decreases in timing performance would become more apparent when probed (1) within a naturalistic scenario and (2) with retrospective duration judgements. Furthermore, as age-related deficits in object–location associations have been reported (Meulenbroek et al., 2010; Shih et al., 2012), and given the higher salience of spatial versus temporal information (Riemer, 2015; Robin et al., 2018), we expected that an age-related effect of attention is more pronounced for the temporal compared with the spatial domain. We thus hypothesised a larger discrepancy between retrospective and prospective judgements for time than for space, and a relative increase in this discrepancy for older compared with younger adults.

## Methods

### Participants

Sixty-five old (37 females; mean age = 71 years, ranging from 65 to 85 years) and 66 young adults (38 females; mean age = 22 years, ranging from 18 to 32 years) participated in the study. Due to technical issues, one older participant did not complete the prospective tasks (cf. section “Experimental protocol and tasks”). Older adults were recruited from the local community in Magdeburg and received monetary compensation. Young adults were recruited from the University of Groningen and participated for course credits (59 %) or monetary compensation (41 %). Participants in Magdeburg and Groningen were German-speaking. The level of education differed between the groups: While the young group consisted entirely of university students who had at least 13 years of education, 20% of the older participants did not reach the official qualification for higher education, (i.e., less than 13 years of school). All participants gave written informed consent to the experimental protocol, which was approved by the ethics committees of the University of Magdeburg (protocol code: 131/14) and the University of Groningen (protocol code: PSY-1819S-0147).

### Experimental stimuli

Participants from each age group were assigned to one of the two different versions of the experimental task. In one version—in the following referred to as the *object version*—presented stimuli were based on 10 image pairs, each consisting of two object images differing in a detail regarding their shape (cf. Figure 1a; stimuli were selected from Berron et al., 2018). For each participant, we created an object sequence consisting of 10 images. For each of these sequences, one image was randomly selected from each of the 10 image pairs and randomly assigned to a specific duration and a specific position on the screen. Thus, each image sequence consisted of the subsequent presentation of 10 images. To contrast temporal and spatial dimensions, every image was presented at one of the six possible positions, arranged equidistantly along the horizontal axis on the screen (−8.5, −5.1, −1.7, 1.7, 5.1, and 8.5 cm relative to the screen centre), and for one of the six possible durations (1.6, 2.4, 3.6, 5.4, 8.1, or 12.15 s). To counteract the influence of the extreme values on performance (as these might have been explicitly encoded), the two extreme values of each dimension (i.e., −8.5/8.5 cm for spatial position and 1.6/12.15 s for duration) were not included in the comparative judgements (cf. section “Experimental protocol and tasks”) and only assigned to one image. Furthermore, extreme durations and extreme positions were never associated with the same image. All other values were used twice (i.e., represented by two images).

Another version of the experimental task—in the following referred to as the *scene version*—consisted of the presentation of a video of an urban scenery, in which 10 events occurred (e.g., the headlights of a car are turned on for a certain duration; cf. Figure 1b). Analogous to the object version, each event was paired with a very similar lure event that did not occur in the video (e.g., differences in the colour of light, whether the left or the right lamppost was illuminated; Figure 1a), and each event lasted for one of the six possible durations that were also used in the object version (i.e., 1.6, 2.4, 3.6, 5.4, 8.1, or 12.15 s). Because the locations of the events were determined by the layout of the visual scene, they were not randomly assigned (as in the object version). However, all events were approximately located at six possible positions along the horizontal axis of the screen (−8.5, −5.1, −1.7, 1.7, 5.1, and 8.5 cm relative to the screen centre), with only one event associated with the two most outward positions and two events associated with each of the four other horizontal positions.

The order of object images (or scene events, respectively) was randomised across participants, and the inter-stimulus interval between two subsequent images (events) was randomly selected from a value between 1 and 3 s. However, within each participant, both the order of images (events) and the inter-stimulus intervals were kept constant so that each individualised object sequence (video) did not change in any aspect. In both task versions, the total duration of the object sequences (videos) ranged between 70 and 78 s.

### Experimental protocol and tasks

Participants were seated in front of a monitor with an effective viewing area of 52 cm × 32.5 cm, at a viewing distance of approximately 50 cm. They were told that they would see the same image sequence (the same video) several times and that they afterwards would have to answer questions regarding the images (events) they see.

In the learning phase, the image sequence (video) was always presented with the instruction to “remember as much as possible” (Figure 1b). A first presentation was directly followed by an open recall test, in which the participants were asked to name as many of the images (events) as they could remember.^1^ After a second presentation of the image sequence (video), participants performed a recognition test (Figure 1c), in which all 10 target images (events) and 10 corresponding lures (cf. Figure 1a) were presented one by one, in random order, in the middle of the screen. The participants then had to judge whether they had seen exactly this image in the sequence (event in the video). Responses were given with the arrow-up key (“yes”) and the arrow-down key (“no”) of a standard keyboard.^2^ A third presentation was again followed by an open recall test and a fourth presentation by another recognition test. After the fifth presentation, participants performed the retrospective time and space judgement tasks. Thus, all participants saw the image sequence (video) five times, before they were alluded to the relevance of the different durations and positions.

For the retrospective judgements on time and space (Figure 1d), two images (events) were presented in the middle of the screen, vertically arranged, and the participants had to indicate which of them had been presented for a longer duration (time judgements) or closer to the right edge of the screen (space judgements). Again, responses were given with up/down arrow keys.

Images (events) associated with the most extreme values of each dimension were excluded from these comparative judgements, for example, regarding time judgements, the image (event) associated with the shortest duration (1.6 s) and the one associated with the longest duration (12.15 s) were excluded. This was done to prevent the performance being determined by an anchoring to one of the extreme values. The remaining eight target images (events) were combined with each other, excluding pairs associated with the same value, resulting in 24 combinations. Each combination was presented twice, with interchanged vertical positions.

For the prospective judgements, participants performed both the time and the space task again, exactly as described above, the only difference being that each task was directly preceded by an additional presentation of the image sequence (video), with the explicit instruction to attend to the durations or locations, respectively. Task order was randomised across, but not within, participants, that is, each participant always performed either first the time and then the space judgements or vice versa.

### Statistical analysis

Data and analysis scripts can be found in OSF (https://osf.io/nfvkw/). Responses associated with reaction times below 200 ms (0.02 %) were discarded as outliers (Woods et al., 2015). Furthermore, time judgement data from two aged participants were excluded from analysis because of error rates above 75 %.

On the basis of the performance in the comparison task, we calculated four psychometric functions per subject, describing the performance in the time and the space task under retrospective and prospective conditions. Slope parameters were fitted with a lower bound of 0. Fitted logistic functions represent the probability of the response “the upper image was longer/more rightward than the lower image,” as a function of the log-transformed^3^ ratio between the durations associated with bottom and top images (for time judgements) or as a function of the difference between the horizontal positions associated with top and bottom images (for space judgements). Precision of judgements was quantified by the slope of the logistic functions.

Data were analysed in R (R Core Team, 2016) and Stan (Carpenter et al., 2017; Stan Development Team, 2017) by fitting multilevel models (2 × 2 × 2 × 2 factorial design) for Bayesian inference using R package *brms* (Bürkner, 2017, 2018) including the within-subjects factors dimension (time vs. space, coded as −.5 and .5) and attentional perspective (retrospective vs. prospective, coded as −.5 and .5) and the between-subjects factors age group (old vs. young, coded as −.5 and .5) and task version (object vs. scene, coded as −.5 and .5). Subjects were included as random factor. As statistics, we report Bayes factors comparing the alternative hypothesis to the null hypothesis (BF_10_), indicating that the data are *BF_10_* times more likely under the alternative compared with the null hypothesis. Values greater than 1 provide support for the alternative hypothesis, whereas values smaller than 1 provide support for the null hypothesis. We used a normally distributed prior with a mean of 0 and a standard deviation of 1.

Working memory capacity was assessed with a delayed word recall task (modelled after the word list recall task of the Consortium to Establish a Registry for Alzheimer’s Disease [CERAD] test battery; Morris et al., 1989). Differences to the original task version consisted in the use of a different set of words (to prevent carry-over effects from previous testing of the same participants). To test whether the results were partially determined by individual differences in working memory capacity, we implemented an alternative model including the performance in the delayed word recall task as an additional factor. However, instead of entering the test score as binary factor indicating whether participants met or failed an age-, sex-, and education-corrected cut-off score, we calculated the individual deviations from the cut-off values. These deviation scores were entered into the model as a continuous variable.

## Results

### Precision of judgements

**Figure 2.**
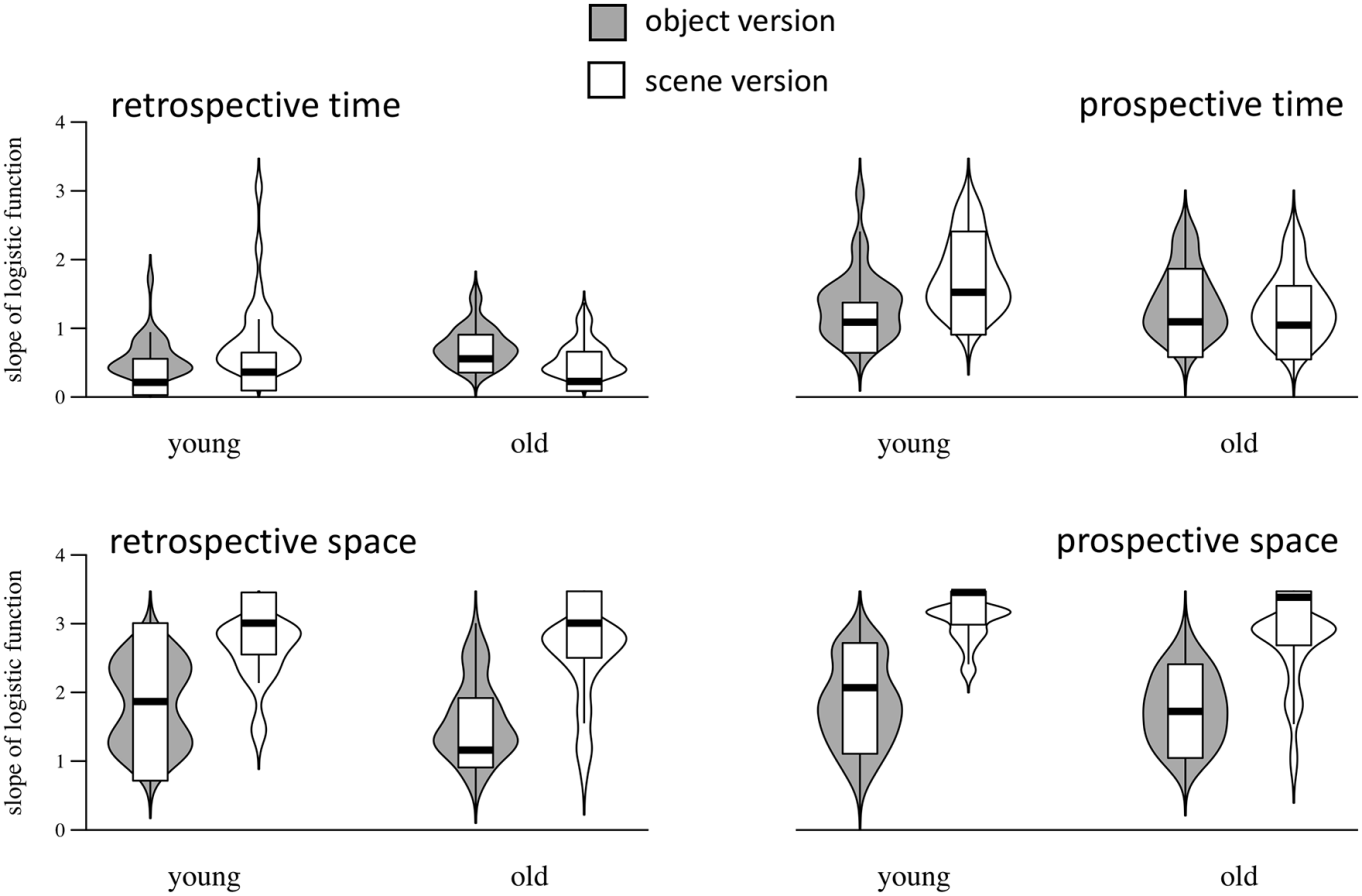
Precision data for retrospective (left column) and prospective judgements (right column), for time (upper row) and space (bottom row). Inner boxes in violin plots represent median and interquartile range.

Results for precision are presented in Figure 2. The main effects of task version, dimension, and attentional perspective reveal that precision was higher for the scene versus the object version (β = 0.67, *SE* = 0.09, BF_10_ >10^3^), for spatial versus temporal judgements (β = 1.42, *SE* = 0.06, BF_10_ >10^3^), and for prospective versus retrospective judgements (β = 0.50, *SE* = 0.06, BF_10_ >10^3^). The main effect of age group did not reach a significant level (β = 0.12, *SE* = 0.09, BF_10_ = 0.22). Furthermore, we found an interaction between dimension and task version, indicating that the advantage of spatial over temporal processing was more pronounced in the scene version (β = 1.05, *SE* = 0.12, BF_10_ >10^3^); an interaction between attentional perspective and dimension, indicating that the benefit of prospective judgements was more pronounced for the time versus the space task (β =-0.65, *SE* = 0.12, BF_10_ >10^3^); and a three-way interaction between age group, dimension, and task version, indicating that, with respect to space judgements, both age groups benefitted equally from an embedding of stimuli within a naturalistic scene, while, with respect to time judgements, older adults did not benefit from the scene version as younger adults did (β =−0.62, *SE* = 0.24, BF_10_ = 6.6). The interaction between age group and attentional perspective was not significant, and the data revealed moderate support in favour of the null hypothesis of no effect (β = 0.12, *SE* = 0.12, BF_10_ = 0.20). All other interactions were not significant (all BF_10_ <0.47 and >0.23).

To test whether these results are influenced by individual differences in working memory capacity, we implemented an alternative model including a main effect and all interactions with performance in the word recall task. None of the interactions incorporating memory performance reached significance (all BF_10_ <0.50). A direct comparison between the base and the alternative model indicated that the latter was not justified (base model: leave-one-out cross-validation information criterion [LOOIC] = 1,199.2, *SE* = 34.4; alternative model: LOOIC = 1,222.1, *SE* = 34.2).

To describe the nature of the three-way interaction, we performed separate models for time and space judgements. In line with our interpretation of the base model, these analyses revealed a significant interaction between age group and task version for time judgements, indicating that older adults did benefit less from the scene version than younger adults (β = 0.46, *SE* = 0.19, BF_10_ = 3.67), but no such interaction for space judgements (β =−0.19, *SE* = 0.26, BF_10_ = 0.34).

### Error rates

**Figure 3.**
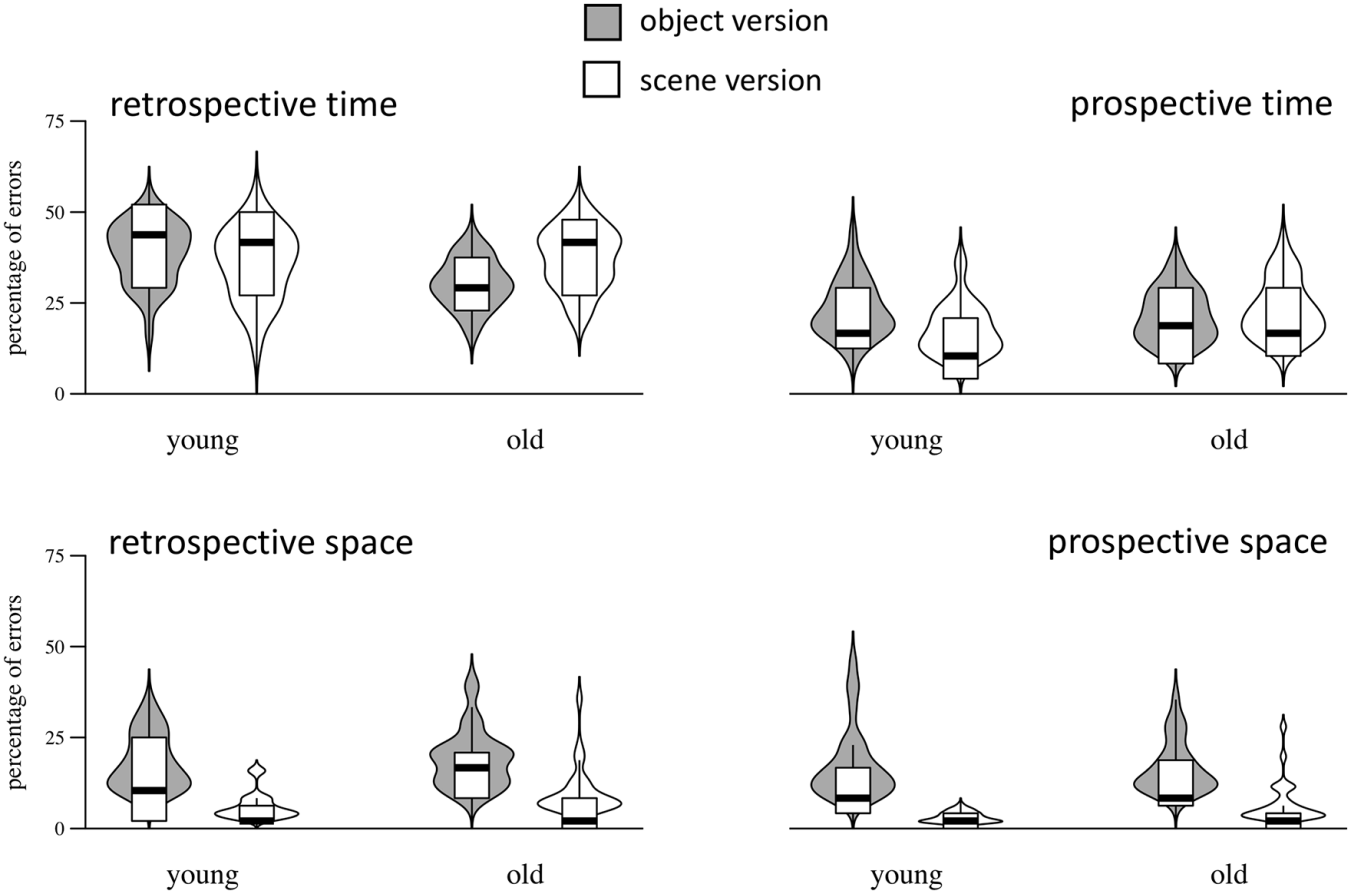
Error rates for retrospective (left column) and prospective judgements (right column), for time (upper row) and space (bottom row). Inner boxes in violin plots represent median and interquartile range.

Results for error rates are presented in Figure 3. Mirroring the results for judgement precision, the percentage of errors was lower for the scene versus the object version (β =−0.06, *SE* = 0.01, BF_10_ >117.5), for spatial versus temporal judgements (β =−0.18, *SE* = 0.01, BF_10_ >10^3^), and for prospective versus retrospective judgements (β =−0.10, *SE* = 0.01, BF_10_ >10^3^). Age groups did not differ in error rates (β = 0.003, *SE* = 0.01, BF_10_ = 0.013). Again, there was an interaction between dimension and task version (β =−0.09, *SE* = 0.02, BF_10_ >10^3^) and between attentional perspective and dimension (β = 0.16, *SE* = 0.02, BF_10_ >10^3^). There was no interaction between age group and attentional perspective (β =−0.04, *SE* = 0.02, BF_10_ = 0.18), nor did the three-way interaction between age group, dimension, and task version reach a significant level (β = 0.09, *SE* = 0.04, BF_10_ = 0.55). An alternative model accounting for individual differences in working memory capacity was not justified (base model: LOOIC =−794.4, *SE* = 37.7; alternative model: LOOIC =−771.0, *SE* = 37.3).

## Discussion

In this study, we investigated timing abilities in older and younger adults and how their timing behaviour is (1) modulated by a naturalistic embedding of duration stimuli and (2) whether retrospective compared with prospective judgements provided differential outcomes. Specifically, we expected that age-related decreases in timing performance are more pronounced when the to-be-timed stimuli (1) are presented within a naturalistic visual scene and (2) were encoded without the participants knowing that their duration will be of relevance as is the case in the retrospective judgements.

Our results demonstrate that older adults, compared with younger ones, react differently to a naturalistic embedding of duration stimuli. While young adults benefit from this manipulation in terms of a higher precision of time judgements and a lower error rate, older adults do not show such a benefit and demonstrate even worse performance (e.g., reduced precision and higher error rates in the retrospective time judgement task; cf. Figures 2 and 3). As this effect remains when individual differences in working memory capacity are taken into account, it does not seem to be driven by such individual differences. With respect to the difference between prospective and retrospective judgements, our results did not reveal an age-related modulation. In both age groups, performance was better for prospective time judgements, and the difference between retrospective and prospective judgements did not increase with age. Although the analysis of error rates revealed a significant interaction between age group and attentional perspective, showing that the ameliorating effect of prospective judgements was slightly smaller for old compared with young adults, this effect was not reflected in judgement precision.

### Retrospective versus prospective judgements

The results of this study suggest that age-related deficits in time perception are independent of the attentional resources allocated to the process of time keeping. The intuitive assumption of a generally superior performance for prospective compared with retrospective time judgements has been confirmed in many studies (Block & Zakay, 1997; El Haj et al., 2013), and the current study did not provide evidence that this effect of attention is modulated by increasing age.

We also found that the increment in performance due to prospective (compared with retrospective) judgements is higher for the temporal domain than for the spatial domain. Although prospective judgements about spatial location were more precise than the retrospective ones, this difference was substantially smaller than for judgements about temporal duration. This finding reveals a critical difference between the attentional resources necessary for the processing of temporal and spatial information: While spatial information is processed almost automatically, the processing of temporal information depends much more on deliberate attention. Note that, also for the spatial domain, we did not find any evidence that this difference is modulated by age.

The absence of significant aging effects on attentional resources devoted to temporal information parallels a related line of research: In a series of studies, Nobre and colleagues investigated the ability to orient attention towards specific moments in time (Coull & Nobre, 2008; Nobre, 2001) and reported that this ability is often preserved in older adults (Chauvin et al., 2016; Heideman et al., 2018, but see Zanto et al., 2011). For example, Heideman et al. (2018) asked old and young participants to determine the orientation of Gabor patches, the temporal onsets of which were cued in each trial. The authors found generally increased perceptual sensitivity and reduced reaction times in response to valid cues, but no difference between the age groups (Heideman et al., 2018).

Our study extends these findings to the domain of temporal durations (as opposed to specific moments in time). Older and young adults were equally capable of directing attentional resources towards the duration of temporal intervals, for both the scene and the object version of our paradigm. This suggests that time-related attentional processes are preserved in older age, with respect to both (1) the ability to orient attention towards specific moments in time and (2) the ability to orient attention towards the duration of temporal intervals.

### Naturalistic versus abstract environments

It has been criticised that paradigms in time perception research often lack ecological validity (Boltz, 2005; Matthews & Meck, 2014; van Rijn, 2018). The experimental tasks often have no relation to everyday behaviours, and the used stimuli often are rather abstract and lacking the complex and dynamic structure of events encountered in real life (Matthews & Meck, 2014). This raises the important question about the degree to which the results from timing experiments in the lab can be generalised to “normal” timing behaviour. Recently, Roseboom et al. (2019) proposed the idea that subjective time is based on the processing of sensory (e.g., visual) content, and hence a direct function of environmental complexity. Especially with respect to age-related changes in timing abilities, the use of artificial scenarios and stimuli outside of any embedding context would diminish the resemblance to the actual difficulties that older adults might experience in their daily lives, and hence reduce the ecological validity of the employed paradigms.

Although there have been some timing studies implementing more naturalistic paradigms and stimuli (Boltz, 2005; Brunec et al., 2017; Riemer et al., 2018; Roseboom et al., 2019; Schlichting et al., 2018; Tobin et al., 2010; van Rijn, 2014), the number of studies directly comparing the effects of naturalistic versus more abstract paradigm versions is rather scarce (Maaß et al., 2021; Thanopoulos et al., 2018). For example, investigating the phenomenon of intentional binding (Haggard et al., 2002), Thanopoulos et al. (2018) found that the tendency to underestimate the temporal interval between a self-initiated action and a subsequent visual stimulus is more pronounced when the nature of the visual stimulus is a meaningful part in a plausible sequence of events (e.g., an image of a hand about to slap a table surface, followed by an image of the same hand touching the surface). This shows that the embedding of experimental stimuli in a naturalistic context can increase their sensitivity to time perception biases.

The differential effect on timing performance of young versus old adults, as it was found in the present study, is a further indication that a naturalistic embedding of stimuli is more effective to target age-related changes in timing behaviour. However, it remains unclear whether the age-related effect of the task version in this study is driven by specific aspects of the naturalistic scene. The effect could be caused by the common semantic context, or by the more dynamic structure of the video scene, or by differences in the visual input (e.g., complexity or content). For example, we know that older adults are more easily distracted by irrelevant information (Gazzaley et al., 2005; Lustig et al., 2007), which might be one reason why their timing performance is impeded by more naturalistic stimuli. Ultimately, the question about the differential impact of influence factors on the age-related effect of a naturalistic embedding of duration stimuli cannot be answered solely on the basis of this study. To enhance our understanding of the mechanisms underlying the temporal processing of naturalistic stimuli, the potential influence factors should be systematically manipulated in future studies.

### The relevance of age-related changes in time perception

Studies in non-human species have identified regions in the medial temporal lobe (MTL)—specifically the hippocampal formation and entorhinal cortex—as core system underlying time-related mnemonic functions (Eichenbaum, 2014; Kraus et al., 2013, 2015; MacDonald et al., 2011), and there is evidence for similar mechanisms in the human brain (Ezzyat & Davachi, 2014; Montchal et al., 2019; Thavabalasingam et al., 2019). As the MTL is known to exhibit elevated levels of tau protein deposition (Harrison et al., 2019; Marks et al., 2017) and age-related neuronal loss (Jack et al., 1998; Raz et al., 2004), particularly in patients with beginning cognitive decline (Braak & Del Tredici, 2015; Fjell et al., 2010), deficits in time perception might potentially serve as behavioural marker for pre-clinical stages of dementia (El Haj & Kapogiannis, 2016; Maaß et al., 2019).

Corroborating this idea, age-related impairments in time perception have often been reported (Block et al., 1998; Mioni et al., 2020; Turgeon et al., 2016; Xu & Church, 2017) and are associated with pathological cognitive decline (Caselli et al., 2009; El Haj et al., 2013; Maaß et al., 2019; Rueda & Schmitter-Edgecombe, 2009). Therefore, the development of reliable paradigms to describe time perception abilities in older populations is of great importance.

With respect to these considerations, our study shows that a naturalistic embedding of duration stimuli might be a useful step towards a thorough description of age-related changes in timing abilities. While the effect of directed attention in prospective time judgements was equal between older and younger adults, older adults reacted differently to the naturalistic embedding of the to-be-timed stimuli. This shows that the claim for a higher ecological validity in time perception experiments (Matthews & Meck, 2014; van Rijn, 2018) is especially relevant for the study of timing abilities in older adults.

It should be noted that in our study older adults sometimes outperformed younger ones, for example, they made less errors in retrospective time judgements. Based on our impression during data acquisition and the participants’ comments, we believe that this might be caused by motivational differences between age groups: More than younger adults, older adults often took personal satisfaction from their participation in the study. Being concerned about cognitive decline, they were specifically motivated to perform well and to demonstrate their preserved capabilities. To account for this phenomenon, our research questions in this study were not focused on general performance levels, but instead on the interactions between critical task manipulations (i.e., a naturalistic embedding of stimuli and a change in attentional perspective).

## Conclusion

Our results have some practical implications for the study of timing abilities in older adults, namely that the nature of the used duration stimuli can affect the results. Specifically, the use of stimuli within of a common semantic context can influence the way in which older adults process the temporal aspects of these stimuli. While young adults show an advantage for durations embedded within a naturalistic context, older adults demonstrate a lack of this advantage. To describe changes in the timing behaviour of older adults, we need to enhance the ecological validity of our experimental tasks. This study suggests the implementation of more naturalistic stimuli as one useful step to achieve this goal.

On a more general level, the results of this study show that our knowledge about human timing behaviour could be informed by a further investigation of the differential effects of naturalistic versus abstract duration stimuli (e.g., by directly comparing these effects in a within-subjects design).

## Notes

1. Incorrect labels that nevertheless demonstrated that the correct object was recalled were regarded as correct (e.g., when the “guitar” was described as “yellow musical instrument”).

2. For the old group, keys were operated by the experimenter (according to the participants’ indication), as older adults often demonstrate problems to withhold motor responses (Kuehn et al., 2018).

3. We log-transformed the duration ratios to ensure an equal absolute difference between the shorter and longer logarithmically spaced durations that were used in this study.
